# How to determine leg dominance: The agreement between self-reported and observed performance in healthy adults

**DOI:** 10.1371/journal.pone.0189876

**Published:** 2017-12-29

**Authors:** Nicky van Melick, Bart M. Meddeler, Thomas J. Hoogeboom, Maria W. G. Nijhuis-van der Sanden, Robert E. H. van Cingel

**Affiliations:** 1 Knee Expert Center Eindhoven, Eindhoven, the Netherlands; 2 Radboud University Medical Center, Research Institute for Health Sciences, IQ healthcare, Nijmegen, the Netherlands; 3 Sport Medisch Centrum Papendal, Arnhem, the Netherlands; Universita degli Studi di Roma 'Foro Italico', ITALY

## Abstract

**Context:**

Since decades leg dominance is suggested to be important in rehabilitation and return to play in athletes with anterior cruciate ligament injuries. However, an ideal method to determine leg dominance in relation to task performance is still lacking.

**Objective:**

To test the agreement between self-reported and observed leg dominance in bilateral mobilizing and unilateral stabilizing tasks, and to assess whether the dominant leg switches between bilateral mobilizing tasks and unilateral stabilizing tasks.

**Design:**

Cross-sectional study.

**Participants:**

Forty-one healthy adults: 21 men aged 36 ± 17 years old and 20 women aged 36 ±15 years old.

**Measurement and analysis:**

Participants self-reported leg dominance in the Waterloo Footedness Questionnaire-Revised (WFQ-R), and leg dominance was observed during performance of four bilateral mobilizing tasks and two unilateral stabilizing tasks. Descriptive statistics and crosstabs were used to report the percentages of agreement.

**Results:**

The leg used to kick a ball had 100% agreement between the self-reported and observed dominant leg for both men and women. The dominant leg in kicking a ball and standing on one leg was the same in 66.7% of the men and 85.0% of the women. The agreement with jumping with one leg was lower: 47.6% for men and 70.0% for women.

**Conclusions:**

It is appropriate to ask healthy adults: “If you would shoot a ball on a target, which leg would you use to shoot the ball?” to determine leg dominance in bilateral mobilizing tasks. However, a considerable number of the participants switched the dominant leg in a unilateral stabilizing task.

## Introduction

Leg dominance is an often discussed factor amongst both healthy and injured athletes. In healthy adults, leg dominance seems to have no influence on knee open kinetic chain proprioception and single-leg postural control [1,2]. However, a systematic review on isokinetic quadriceps and hamstring strength and single-leg hop performance found non-significant but clinical important differences in the performance of the dominant leg compared to the non-dominant leg, with the dominant leg scoring higher values for all these tasks [3]. Furthermore, leg dominance appears to play a role in the etiology of anterior cruciate ligament injuries, because female recreational soccer players and skiers are more likely to injure their non-dominant leg, whereas males tend to injure their dominant leg [4–6].

In the above studies, different methods to determine leg dominance are used, thus an ideal method to determine leg dominance is still lacking [7,8]. In 1998, Peters defined the dominant leg as ‘the leg used in order to manipulate an object or to lead out in movement’ [9]. This automatically leads to the definition of the non-dominant leg: ‘the leg which performs the stabilizing or supporting role’ [9]. Several footedness questionnaires have been developed over time in order to determine leg dominance [10,11]. These questionnaires have frequently been used by other authors, however hardly any statements on the correlation between the self-reported leg dominance in a questionnaire and the actual observed performance of those tasks have been made. To our knowledge, only Hart and Gabbard (1998) investigated this relationship and stated that there is a strong agreement (98%) for right-footers between leg dominance indicated by responses in a questionnaire and leg dominance demonstrated on two tasks [12]. For left-footers this agreement was 84%. These tasks were rolling a golf ball around a circle as quickly and accurate as possible with one foot while seated, and drawing initials in a sandbox using one foot while seated [12]. It should be noted that the tasks used in this study are not very common in daily life. Besides, there is no supporting leg in these seated tasks, which makes it unclear whether the definition of Peters can be used in determining the dominant leg in sport activities.

One of the uncertainties in determining leg dominance is the fact that literature reports variation in leg dominance between different types of tasks [3]. In bilateral mobilizing tasks, such as kicking a ball while standing, both legs are involved. In unilateral stabilizing tasks, however, such as standing on one leg, merely only one leg is active. In this case the standing leg is the dominant leg, according to Peters [9]. Hart and Gabbard (1997) claimed that the dominant leg in bilateral mobilizing tasks, in general, is also the dominant leg in unilateral stabilizing tasks, thus the standing leg will switch [13]. However, in their own study, only 62% of the right-handed and 44% of the left-handed participants switched the standing leg in the bilateral task compared to the unilateral task, so apparently there is not one dominant leg for all tasks [13].

The first aim of this study is to determine the most accurate question to ask for leg dominance based on the agreement between the self-reported leg dominance using the Waterloo Footedness Questionnaire-Revised (WFQ-R) questionnaire [11] and the observed leg dominance in four bilateral mobilizing tasks and two unilateral stabilizing tasks. The second aim of this study is to retest the phenomenon described by Hart and Gabbard, that the standing leg is switched between a bilateral mobilizing task and a unilateral stabilizing task to remain the same dominant leg.

## Materials and methods

### Participants

All participants in this study were healthy volunteers (students or teachers at the Radboud university medical center), recruited by personal contacts of the authors. They were unaware of the actual purpose of this study and were told that this study investigated their lower extremity coordination. The inclusion criteria were: age between 18 and 65 years old, practice of symmetrical sports (e.g. running, cycling, swimming, rowing) or sports which involve the lower extremities only (e.g. soccer) or people who do not practice any type of sport. Participants that practiced sports in which the upper extremity is predominantly used (e.g. handball, tennis, volleyball) were excluded, because of the introduction of a possible bias as stated by Peters, who mentioned that in athletics, the choice of arm usually influences the choice of the leg [9]. Other exclusion criteria were surgery to one or both legs in the past three years, a back or lower extremity injury at the moment of testing, the use of medication which influences balance, and the presence of any disease which affects balance or coordination.

Forty-one healthy adults were eligible for inclusion: 21 men aged 35.8 ± 16.5 years old and 20 women aged 36.1 ±15.2 years old. 90% of them were right-handed, which is comparable to the world population [14]. All participants agreed to take part in this study and gave their written informed consent to their inclusion in this study. This study was approved by the Medical Ethics Committee Arnhem/Nijmegen (registration number 2017–3373).

### Test procedures

#### Self-reported leg dominance

**Fig 1:**
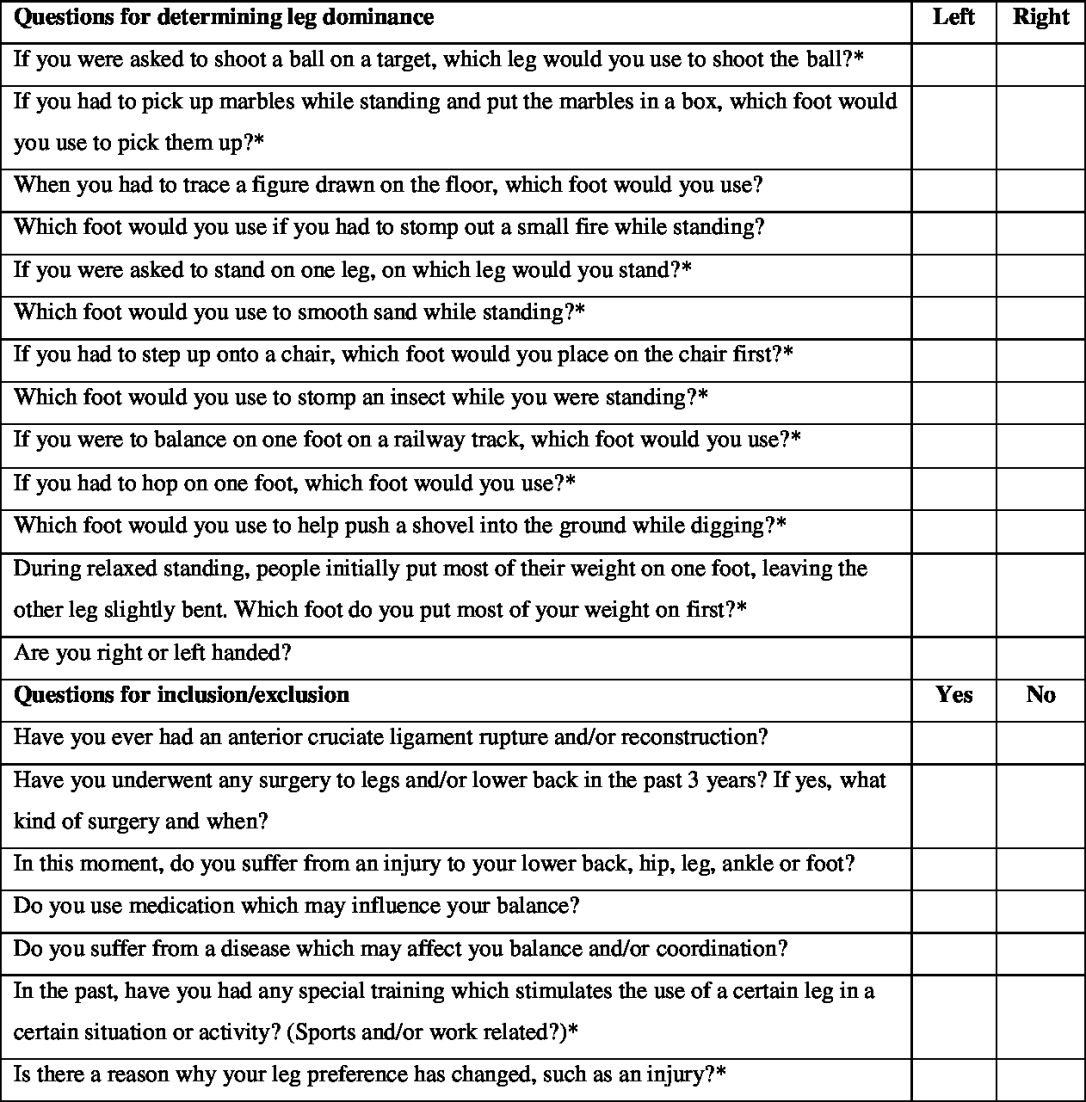
Leg domiance questionaire used in this study. The original questions of the WFQ-R are marked with a *.

The WFQ-R was used in order to identify the participant’s own experienced leg dominance [11]. To this 12-item questionnaire, eight questions were added based on other tasks previously described for determining leg dominance (Fig 1) [15]. The participants were asked to complete the questionnaire first, before the tasks were performed, and they were unaware of the fact that some tasks, which were part of the questionnaire, had to be performed later on. To avoid recollection of questionnaire items when performing the tasks as much as possible, a higher number of tasks was used in the questionnaire than those that actually had to be performed.

#### Observed leg dominance

Only six of the tasks were executed by the participants: kicking a ball at a target placed four meters away, picking up five marbles which are arranged in a vertical line and putting them in a box by using one foot while standing, stomping out an imaginary fire displayed on a sheet of paper using one foot while standing, tracing the shape of a house using one foot while standing, standing on one leg on an unstable foam surface with eyes closed, and jumping as far as possible with one leg. The first four tasks were labeled as reliable bilateral mobilizing tasks by Schneiders et al. (kappa’s between 0.61 and 0.88) and were recommended to be used in a test battery to determine leg dominance [15]. Standing on one leg and jumping with one leg were added as unilateral stabilizing tasks in order to assess the dominant leg in these types of tasks for the second aim.

All tasks were performed three times in a randomly assigned order, without any footwear. For each task, the dominant leg was recorded as the dominant leg used in at least two out of three repetitions. Besides, the stability within a tasks was registered. A task was named stable when all three repetitions were performed with the same leg as the dominant leg.

For each task, the starting position for the participant was marked with a piece of tape on the floor. Feet were placed at hip width apart and parallel to each other. In order to prevent any external influence on the selected leg to perform the task, the objects used for the tasks (such as a ball or marbles) were placed on marked positions midway between the feet. The researcher made no mention regarding limb choice [12, 15]. Additionally, during each task, a supplementary cognitive calculating task was given, which started prior to the execution of the task and lasted until task execution was completed. This cognitive distraction was implemented to draw away the focus on the selected leg used to perform the task [16].

### Statistical analysis

All data were analyzed using SPSS 19.0.0. Descriptive statistics was used to describe the percentage of participants choosing the right leg in the self-reported and observed leg dominance tasks. In addition, the percentage of agreement in leg choice between the self-reported and observed choice during task execution was reported with Crosstabs. Subsequently, the bilateral mobilizing task with the highest agreement was used to determine the most accurate question to ask for leg dominance.

The bilateral mobilizing task with the highest percentage of agreement was compared to the unilateral stabilizing tasks to investigate our second study aim.

## Results

The results of the analysis of the first aim are displayed in Table 1 for both bilateral mobilizing tasks and unilateral stabilizing tasks. Only for kicking the ball, the observed leg dominance 100% matches the self-reported leg dominance and is a stable task for both men and women. Therefore, this bilateral mobilizing task was used to compare the dominant leg with the unilateral stabilizing tasks for the second study aim. The results of this analysis are shown in Table 2. The agreement with standing on one leg is the highest, with 66.7% for men and 85.0% for women. The agreement with jumping with one leg is lower: 47.6% for men and 70.0% for women.

**Table 1:** Percentages of agreement between self-reported and observed leg dominance and task stability for the four bilateral mobilizing tasks and two unilateral stabilizing tasks.

Task		Men	Women
**Kicking ball**	Self-reported (% using right leg)	95.2	100.0
	Observed (% using right leg)	95.2	100.0
	Agreement (%)	100.0	100.0
	Task stability (% of participants with stable task)	95.2	100.0
**Picking marbles**	Self-reported (% using right leg)	85.7	95.0
	Observed (% using right leg)	90.5	100.0
	Agreement (%)	95.2	95.0
	Task stability (% of participants with stable task)	90.5	100.0
**Tracing shape**	Self-reported (% using right leg)	81.0	100.0
	Observed (% using right leg)	81.0	100.0
	Agreement (%)	90.5	100.0
	Task stability (% of participants with stable task)	90.5	100.0
**Stomping out fire**	Self-reported (% using right leg)	81.0	100.0
	Observed (% using right leg)	95.2	100.0
	Agreement (%)	90.5	100.0
	Task stability (% of participants with stable task)	95.2	100.0
**Standing one leg**	Self-reported (% using right leg)	71.4	85.0
	Observed (% using right leg)	76.2	80.0
	Agreement (%)	85.7	95.0
	Task stability (% of participants with stable task)	85.7	90.0
**Jumping one leg**	Self-reported (% using right leg)	61.9	65.0
	Observed (% using right leg)	52.4	70.0
	Agreement (%)	71.4	85.0
	Task stability (% of participants with stable task)	81.0	85.0

**Table 2:** Percentage of agreement between the dominant leg of the best bilateral mobilizing task (kicking a ball) with the unilateral stabilizing tasks.

	Men	Women
**Standing one leg**	66.7	85.0
**Jumping one leg**	47.6	70.0

## Discussion

This study on leg dominance examined two research questions in healthy adults. The first aim was to determine the most accurate question to ask for leg dominance based on the agreement between the self-reported and observed leg dominance in four bilateral mobilizing tasks and two unilateral stabilizing tasks. Our results show that the question “If you would shoot a ball on a target, which leg would you use to shoot the ball?” showed the highest agreement (100% for both men and women) and was the most stable task (95.2% for men and 100.0% for women) of the bilateral mobilizing tasks, Of the unilateral stabilizing tasks standing on one leg showed the highest agreement (85.7% for men and 95.0% for women) and also was the most stable task (85.7% for men and 90.0% for women). Only one study previously made a statement about the correlation between self-reported and observed leg dominance. According to Hart and Gabbard, 98% of right-footers and 84% of left-footers showed an agreement between the preferred leg in unilateral mobilizing (seated) tasks [12]. Right-handed people performed activities more consistently with one lower extremity when compared with left-handed adults [17]. The results in our study for kicking a ball are more conclusive, as this is 100% for both right- and left-handed participants. A remark in the study by Hart and Gabbard is that unilateral mobilizing tasks have been used in order to determine the dominant leg, whereas in our study bilateral mobilizing tasks have been used. The tasks used in our study presumably require more dexterity and accuracy compared to the unilateral mobilizing tasks and may be executed using different spinal pathways, possibly impeding a direct comparison [18]. Moreover, it should be noted that unilateral mobilizing tasks are hardly present during daily life or in sports and therefore show a more unstable pattern in leg preference than tasks that are more common [19]. This makes the tasks used by Hart and Gabbard less applicable in a general athletic population. We postulate that a more automatically performed task, with no or a minor motor learning effect, could provide a better agreement between the question asked which leg will be used and the actual task performance.

The second aim of this study was to retest the phenomenon described by Hart and Gabbard, that the standing leg is switched between a bilateral mobilizing task and a unilateral stabilizing task to remain the same dominant leg [13]. The agreement in Hart and Gabbard’s study was 62% in a right-handed population and 44% in a left-handed population. In our opinion, this percentage is low. The results from our study show a higher percentage of participants (66.7% for men and 85.0% for women) who have the same dominant leg when comparing kicking a ball and standing on one leg. However, jumping on one leg more resembles the need of athletes compared to standing on one leg. When comparing kicking a ball and jumping on one leg, more than 50% of the men and 30% of the women had a different dominant leg for both tasks. These numbers are similar to the percentage of Hart and Gabbard. With respect to these findings, there still is an amount of variability between the dominant leg in the bilateral mobilizing and unilateral stabilizing context. This task dependency is previously mentioned by other authors. A strong preference for one foot in mobilization tasks is contrasted to large interindividual variability and weak foot preference in stabilization tasks, as can be seen when task stability is compared between bilateral mobilizing tasks and unilateral stabilizing tasks (Table 1) [15,19,20].

Our study results may have implications for lower limb injury rehabilitation and return to play. For example, the Limb Symmetry Index (LSI) is a popular tool for monitoring progression trough rehabilitation and determining the moment athletes can return to play after lower limb injuries [21–23]. The LSI is used to compare the operated to the non-operated leg when measuring quantitative components of movement like strength tests or hop tests [21,24]. If the operated leg is compared to the non-operated leg, the LSI does not take leg dominance into account [25]. Nowadays there is still a debate whether it is relevant to discriminate between the dominant and non-dominant leg in lower limb rehabilitation [21,25–27]. However, literature suggests the LSI should be above 100% when calculated as the value of the dominant leg divided by the value of the non-dominant leg to determine safe return to play (McGrath) [3,28–30]. Future research should indicate whether the dominant leg has a superior performance compared to the non-dominant leg and what the LSI values for safe return to play should be.

### Strengths and limitations

A strength of this study is that we also chose bilateral mobilizing tasks to answer the first aim. Tasks, like kicking a ball, are related to daily life of many athletes and therefore are more stable in foot preference than unilateral mobilizing tasks as used by Hart and Gabbard [12]. However, in this study we only examined healthy adults.

A limitation of this study is that only 10% of the study population was left-handed. Therefore, the results for the left-handed participants should be interpreted with caution. The proportion right- and left handed adults in this study, however, is comparable to the world population [14].

## Conclusions

To determine leg dominance in healthy adults, the question “If you would shoot a ball on a target, which leg would you use to shoot the ball?” is accurate for bilateral mobilizing tasks. The dominant leg in this bilateral mobilizing task is also the dominant leg in a unilateral stabilizing task (e.g. jumping on one leg) in about 50% of men and 70% of women.

## Supporting information

S1 File Data set observed leg dominance. (PDF)

S2 File Data set observed vs self-reported leg dominance. (PDF)
