# Supplementary material for: How to determine leg dominance: The agreement between self-reported and observed performance in healthy adults
Source: PLoS One. 2017 Dec 29;12(12):e0189876. doi: 10.1371/journal.pone.0189876 (PMC5747428; doi:10.1371/journal.pone.0189876)
Supplement: S2 File — (PDF) [file pone.0189876.s002.pdf]

| Seks | Age | Standing observed | Standing self-reported | Standing same | Jumping observed | Jumping self-reported | Jumping same | Kicking observed | Kicking self-reported | Kicking same | Jumping-kicking same | Standing-kicking same | Marbles observed | Marbles self-reported | Marbles same | Shape observed | Shape self-reported | Shape same | Fire observed | Fire self-reported | Fire same |
|---|---|---|---|---|---|---|---|---|---|---|---|---|---|---|---|---|---|---|---|---|---|
| Female | 20 | Right | Right | Yes | Right | Right | Yes | Right | Right | Yes | Yes | Yes | Right | Right | Yes | Right | Right | Yes | Right | Right | Yes |
| Female | 55 | Right | Right | Yes | Left | Left | Yes | Right | Right | Yes | No | Yes | Right | Right | Yes | Right | Right | Yes | Right | Right | Yes |
| Female | 44 | Right | Left | No | Right | Right | Yes | Right | Right | Yes | Yes | Yes | Right | Right | Yes | Right | Right | Yes | Right | Right | Yes |
| Female | 20 | Right | Right | Yes | Right | Left | No | Right | Right | Yes | Yes | Yes | Right | Right | Yes | Right | Right | Yes | Right | Right | Yes |
| Female | 50 | Left | Left | Yes | Left | Left | Yes | Right | Right | Yes | No | No | Right | Right | Yes | Right | Right | Yes | Right | Right | Yes |
| Female | 59 | Right | Right | Yes | Right | Right | Yes | Right | Right | Yes | Yes | Yes | Right | Right | Yes | Right | Right | Yes | Right | Right | Yes |
| Female | 19 | Right | Right | Yes | Right | Right | Yes | Right | Right | Yes | Yes | Yes | Right | Right | Yes | Right | Right | Yes | Right | Right | Yes |
| Female | 27 | Right | Right | Yes | Right | Right | Yes | Right | Right | Yes | Yes | Yes | Right | Right | Yes | Right | Right | Yes | Right | Right | Yes |
| Female | 68 | Right | Right | Yes | Left | Right | No | Right | Right | Yes | No | Yes | Right | Right | Yes | Right | Right | Yes | Right | Right | Yes |
| Female | 32 | Left | Left | Yes | Left | Left | Yes | Right | Right | Yes | No | No | Right | Right | Yes | Right | Right | Yes | Right | Right | Yes |
| Female | 25 | Right | Right | Yes | Right | Left | No | Right | Right | Yes | Yes | Yes | Right | Right | Yes | Right | Right | Yes | Right | Right | Yes |
| Female | 27 | Right | Right | Yes | Right | Right | Yes | Right | Right | Yes | Yes | Yes | Right | Right | Yes | Right | Right | Yes | Right | Right | Yes |
| Female | 50 | Right | Right | Yes | Right | Right | Yes | Right | Right | Yes | Yes | Yes | Right | Left | No | Right | Right | Yes | Right | Right | Yes |
| Female | 41 | Right | Right | Yes | Right | Right | Yes | Right | Right | Yes | Yes | Yes | Right | Right | Yes | Right | Right | Yes | Right | Right | Yes |
| Female | 29 | Right | Right | Yes | Left | Left | Yes | Right | Right | Yes | No | Yes | Right | Right | Yes | Right | Right | Yes | Right | Right | Yes |
| Female | 37 | Right | Right | Yes | Right | Right | Yes | Right | Right | Yes | Yes | Yes | Right | Right | Yes | Right | Right | Yes | Right | Right | Yes |
| Female | 23 | Left | Left | Yes | Left | Left | Yes | Right | Right | Yes | No | No | Right | Right | Yes | Right | Right | Yes | Right | Right | Yes |
| Female | 19 | Right | Right | Yes | Right | Right | Yes | Right | Right | Yes | Yes | Yes | Right | Right | Yes | Right | Right | Yes | Right | Right | Yes |
| Female | 24 | Right | Right | Yes | Right | Right | Yes | Right | Right | Yes | Yes | Yes | Right | Right | Yes | Right | Right | Yes | Right | Right | Yes |
| Female | 52 | Right | Right | Yes | Right | Right | Yes | Right | Right | Yes | Yes | Yes | Right | Right | Yes | Right | Right | Yes | Right | Right | Yes |
| Male | 57 | Right | Right | Yes | Right | Right | Yes | Right | Right | Yes | Yes | Yes | Right | Right | Yes | Left | Left | Yes | Right | Left | No |
| Male | 20 | Right | Right | Yes | Right | Right | Yes | Right | Right | Yes | Yes | Yes | Right | Right | Yes | Right | Right | Yes | Right | Right | Yes |
| Male | 41 | Right | Right | Yes | Left | Right | No | Right | Right | Yes | No | Yes | Right | Right | Yes | Right | Left | No | Right | Right | Yes |
| Male | 21 | Left | Right | No | Left | Left | Yes | Right | Right | Yes | No | No | Right | Right | Yes | Right | Right | Yes | Right | Right | Yes |
| Male | 49 | Right | Right | Yes | Right | Right | Yes | Right | Right | Yes | Yes | Yes | Right | Right | Yes | Right | Right | Yes | Right | Right | Yes |
| Male | 39 | Left | Right | No | Right | Right | Yes | Right | Right | Yes | Yes | No | Right | Right | Yes | Left | Right | No | Right | Right | Yes |
| Male | 63 | Right | Right | Yes | Right | Right | Yes | Right | Right | Yes | Yes | Yes | Right | Right | Yes | Right | Right | Yes | Right | Right | Yes |
| Male | 62 | Right | Right | Yes | Right | Right | Yes | Right | Right | Yes | Yes | Yes | Right | Right | Yes | Right | Right | Yes | Right | Right | Yes |
| Male | 23 | Right | Right | Yes | Right | Left | No | Left | Left | Yes | No | No | Left | Left | Yes | Left | Left | Yes | Left | Left | Yes |
| Male | 68 | Right | Right | Yes | Left | Right | No | Right | Right | Yes | No | Yes | Right | Right | Yes | Right | Right | Yes | Right | Right | Yes |
| Male | 22 | Right | Right | Yes | Left | Right | No | Right | Right | Yes | No | Yes | Right | Left | No | Left | Left | Yes | Right | Right | Yes |
| Male | 48 | Right | Right | Yes | Right | Right | Yes | Right | Right | Yes | Yes | Yes | Right | Right | Yes | Right | Right | Yes | Right | Right | Yes |
| Male | 20 | Right | Right | Yes | Left | Left | Yes | Right | Right | Yes | No | Yes | Right | Right | Yes | Right | Right | Yes | Right | Right | Yes |
| Male | 26 | Right | Right | Yes | Right | Right | Yes | Right | Right | Yes | Yes | Yes | Right | Right | Yes | Right | Right | Yes | Right | Right | Yes |
| Male | 22 | Right | Right | Yes | Left | Left | Yes | Right | Right | Yes | No | Yes | Right | Right | Yes | Right | Right | Yes | Right | Right | Yes |
| Male | 33 | Left | Left | Yes | Left | Left | Yes | Right | Right | Yes | No | No | Right | Right | Yes | Right | Right | Yes | Right | Right | Yes |
| Male | 23 | Left | Left | Yes | Left | Right | No | Right | Right | Yes | No | No | Right | Right | Yes | Right | Right | Yes | Right | Right | Yes |
| Male | 23 | Right | Right | Yes | Right | Left | No | Right | Right | Yes | Yes | Yes | Left | Left | Yes | Right | Right | Yes | Right | Right | Yes |
| Male | 20 | Right | Left | No | Right | Right | Yes | Right | Right | Yes | Yes | Yes | Right | Right | Yes | Right | Right | Yes | Right | Right | Yes |
| Male | 25 | Left | Left | Yes | Left | Left | Yes | Right | Right | Yes | No | No | Right | Right | Yes | Right | Right | Yes | Right | Left | No |
| Male | 47 | Left | Left | Yes | Left | Left | Yes | Right | Right | Yes | No | No | Right | Right | Yes | Right | Right | Yes | Right | Right | Yes |

**Captions**
Standing = Standing on one leg
Jumping = jumping with one leg
Kicking = kicking a ball
Marbles = picking up marbles
Shape = tracing the shape of a house
Fire = stomping out an imaginary fire
… observed = dominant leg for this task
… self-reported = self-reported dominant leg on questionnaire
… same = Is there an agreement between the observed and self-reported leg dominance?
Jumping-kicking same = Is there an agreement between the dominant leg for jumping with one leg and kicking a ball?
Standing-kicking same = Is there an agreement between the dominant leg for standing on one leg and kicking a ball?
