# Supplementary material for: How to determine leg dominance: The agreement between self-reported and observed performance in healthy adults
Source: PLoS One. 2017 Dec 29;12(12):e0189876. doi: 10.1371/journal.pone.0189876 (PMC5747428; doi:10.1371/journal.pone.0189876)
Supplement: S1 File — (PDF) [file pone.0189876.s001.pdf]

	Standing1	Standing2	Standing3	Standing observed	Jumping1	Jumping2	Jumping3	Jumping observed	Kicking1	Kicking2	Kicking3	Kicking observed	Marbles1	Marbles2	Marbles3	Marbles observed	Shape1	Shape2	Shape3	Shape observed	Fire1	Fire2	Fire3	Fire observed
Female	Right	Right	Right	Right	Right	Right	Right	Right	Right	Right	Right	Right	Right	Right	Right	Right	Right	Right	Right	Right	Right	Right	Right	Right
Female	Right	Left	Right	Right	Left	Left	Left	Left	Right	Right	Right	Right	Right	Right	Right	Right	Right	Right	Right	Right	Right	Right	Right	Right
Female	Right	Right	Right	Right	Right	Right	Right	Right	Right	Right	Right	Right	Right	Right	Right	Right	Right	Right	Right	Right	Right	Right	Right	Right
Female	Right	Right	Right	Right	Right	Right	Right	Right	Right	Right	Right	Right	Right	Right	Right	Right	Right	Right	Right	Right	Right	Right	Right	Right
Female	Left	Left	Left	Left	Left	Left	Left	Left	Right	Right	Right	Right	Right	Right	Right	Right	Right	Right	Right	Right	Right	Right	Right	Right
Female	Right	Right	Right	Right	Right	Right	Right	Right	Right	Right	Right	Right	Right	Right	Right	Right	Right	Right	Right	Right	Right	Right	Right	Right
Female	Right	Right	Right	Right	Right	Right	Right	Right	Right	Right	Right	Right	Right	Right	Right	Right	Right	Right	Right	Right	Right	Right	Right	Right
Female	Right	Right	Right	Right	Right	Right	Right	Right	Right	Right	Right	Right	Right	Right	Right	Right	Right	Right	Right	Right	Right	Right	Right	Right
Female	Right	Right	Right	Right	Right	Left	Left	Left	Right	Right	Right	Right	Right	Right	Right	Right	Right	Right	Right	Right	Right	Right	Right	Right
Female	Left	Left	Left	Left	Left	Left	Left	Left	Right	Right	Right	Right	Right	Right	Right	Right	Right	Right	Right	Right	Right	Right	Right	Right
Female	Right	Right	Right	Right	Right	Right	Right	Right	Right	Right	Right	Right	Right	Right	Right	Right	Right	Right	Right	Right	Right	Right	Right	Right
Female	Right	Right	Right	Right	Right	Right	Right	Right	Right	Right	Right	Right	Right	Right	Right	Right	Right	Right	Right	Right	Right	Right	Right	Right
Female	Right	Right	Right	Right	Right	Right	Right	Right	Right	Right	Right	Right	Right	Right	Right	Right	Right	Right	Right	Right	Right	Right	Right	Right
Female	Right	Right	Right	Right	Left	Right	Right	Right	Right	Right	Right	Right	Right	Right	Right	Right	Right	Right	Right	Right	Right	Right	Right	Right
Female	Right	Right	Right	Right	Left	Left	Left	Left	Right	Right	Right	Right	Right	Right	Right	Right	Right	Right	Right	Right	Right	Right	Right	Right
Female	Right	Right	Right	Right	Right	Left	Right	Right	Right	Right	Right	Right	Right	Right	Right	Right	Right	Right	Right	Right	Right	Right	Right	Right
Female	Left	Left	Left	Left	Left	Left	Left	Left	Right	Right	Right	Right	Right	Right	Right	Right	Right	Right	Right	Right	Right	Right	Right	Right
Female	Right	Right	Right	Right	Right	Right	Right	Right	Right	Right	Right	Right	Right	Right	Right	Right	Right	Right	Right	Right	Right	Right	Right	Right
Female	Right	Right	Right	Right	Right	Right	Right	Right	Right	Right	Right	Right	Right	Right	Right	Right	Right	Right	Right	Right	Right	Right	Right	Right
Female	Right	Left	Right	Right	Right	Right	Right	Right	Right	Right	Right	Right	Right	Right	Right	Right	Right	Right	Right	Right	Right	Right	Right	Right
Male	Right	Right	Right	Right	Right	Right	Right	Right	Right	Right	Right	Right	Right	Right	Right	Right	Left	Left	Left	Left	Right	Right	Right	Right
Male	Right	Right	Right	Right	Right	Right	Right	Right	Right	Right	Right	Right	Right	Right	Right	Right	Right	Right	Right	Right	Right	Right	Right	Right
Male	Right	Right	Right	Right	Left	Left	Left	Left	Right	Right	Right	Right	Right	Right	Right	Right	Right	Right	Right	Right	Right	Right	Right	Right
Male	Right	Left	Left	Left	Left	Left	Left	Left	Right	Right	Right	Right	Right	Right	Right	Right	Right	Right	Right	Right	Right	Right	Right	Right
Male	Right	Right	Right	Right	Right	Right	Right	Right	Right	Right	Right	Right	Right	Right	Right	Right	Right	Right	Right	Right	Right	Right	Right	Right
Male	Left	Left	Left	Left	Right	Left	Right	Right	Right	Left	Right	Right	Right	Left	Right	Right	Right	Left	Left	Left	Left	Right	Right	Right
Male	Right	Right	Right	Right	Right	Right	Right	Right	Right	Right	Right	Right	Right	Right	Right	Right	Right	Right	Right	Right	Right	Right	Right	Right
Male	Right	Right	Right	Right	Right	Right	Right	Right	Right	Right	Right	Right	Right	Right	Right	Right	Right	Right	Right	Right	Right	Right	Right	Right
Male	Left	Right	Right	Right	Right	Right	Right	Right	Left	Left	Left	Left	Left	Left	Left	Left	Left	Left	Left	Left	Left	Left	Left	Left
Male	Right	Right	Right	Right	Right	Left	Left	Left	Right	Right	Right	Right	Right	Right	Right	Right	Right	Right	Right	Right	Right	Right	Right	Right
Male	Right	Right	Right	Right	Right	Left	Left	Left	Right	Right	Right	Right	Right	Right	Right	Right	Left	Left	Left	Left	Right	Right	Right	Right
Male	Right	Right	Right	Right	Right	Right	Right	Right	Right	Right	Right	Right	Right	Right	Right	Right	Right	Right	Right	Right	Right	Right	Right	Right
Male	Right	Right	Right	Right	Left	Left	Left	Left	Right	Right	Right	Right	Right	Right	Right	Right	Right	Right	Right	Right	Right	Right	Right	Right
Male	Right	Right	Right	Right	Right	Right	Right	Right	Right	Right	Right	Right	Right	Right	Right	Right	Right	Right	Right	Right	Right	Right	Right	Right
Male	Right	Right	Right	Right	Right	Right	Left	Left	Right	Right	Right	Right	Right	Right	Right	Right	Right	Right	Right	Right	Right	Right	Right	Right
Male	Left	Left	Left	Left	Left	Left	Left	Left	Right	Right	Right	Right	Right	Left	Right	Right	Right	Right	Right	Right	Right	Right	Right	Right
Male	Left	Left	Left	Left	Left	Left	Left	Left	Right	Right	Right	Right	Right	Right	Right	Right	Right	Right	Right	Right	Right	Right	Right	Right
Male	Right	Right	Right	Right	Right	Right	Right	Right	Right	Right	Right	Right	Left	Left	Left	Left	Right	Right	Right	Right	Right	Right	Right	Right
Male	Right	Right	Right	Right	Right	Right	Right	Right	Right	Right	Right	Right	Right	Right	Right	Right	Right	Right	Right	Right	Right	Right	Right	Right
Male	Left	Left	Left	Left	Left	Left	Left	Left	Right	Right	Right	Right	Right	Right	Right	Right	Right	Right	Right	Right	Right	Right	Right	Right
Male	Left	Right	Left	Left	Left	Left	Left	Left	Right	Right	Right	Right	Right	Right	Right	Right	Right	Right	Right	Right	Right	Right	Right	Right

Captions

Standing ... = Standing on one leg

Jumping … = jumping with one leg

Kicking ... = kicking a ball

Marbles … = picking up marbles

Shape … = tracing the shape of a house

Fire … = stomping out an imaginary fire

…1 = dominant leg the first time task is performed

…2 = dominant leg the second time task is performed

…3 = dominant leg the third time task is performed

… observed = observed dominant leg for this task
